# Statistical Enrichment of Epigenetic States Around Triplet Repeats that Can Undergo Expansions

**DOI:** 10.3389/fnins.2016.00092

**Published:** 2016-03-08

**Authors:** Alexandra Essebier, Patricia Vera Wolf, Minh Duc Cao, Bernard J. Carroll, Sureshkumar Balasubramanian, Mikael Bodén

**Affiliations:** ^1^School of Chemistry and Molecular Biosciences, The University of QueenslandSt Lucia, QLD, Australia; ^2^School of Biological Sciences, Monash UniversityMelbourne, VIC, Australia

**Keywords:** short tandem repeat, bioinformatics, epigenetics, histone modification, DNA methylation, genome sequence

## Abstract

More than 30 human genetic diseases are linked to tri-nucleotide repeat expansions. There is no known mechanism that explains repeat expansions in full, but changes in the epigenetic state of the associated locus has been implicated in the disease pathology for a growing number of examples. A comprehensive comparative analysis of the genomic features associated with diverse repeat expansions has been lacking. Here, in an effort to decipher the propensity of repeats to undergo expansion and result in a disease state, we determine the genomic coordinates of tri-nucleotide repeat tracts at base pair resolution and computationally establish epigenetic profiles around them. Using three complementary statistical tests, we reveal that several epigenetic states are enriched around repeats that are associated with disease, even in cells that do not harbor expansion, relative to a carefully stratified background. Analysis of over one hundred cell types reveals that epigenetic states generally tend to vary widely between genic regions and cell types. However, there is qualified consistency in the epigenetic signatures of repeats associated with disease suggesting that changes to the chromatin and the DNA around an expanding repeat locus are likely to be similar. These epigenetic signatures may be exploited further to develop models that could explain the propensity of repeats to undergo expansions.

## 1. Introduction

Expansion of short tandem repeats (STRs) in human genes has been shown to cause a range of debilitating neurological diseases (Kovtun and McMurray, 2008; Dion and Wilson, 2009; Cohen-Carmon and Meshorer, 2012). Repeat expansions can be observed in both germline and somatic tissues, but the factors that underlie the expansion of short tandem repeats in general are poorly understood. Changes in epigenetic status surrounding repeat expansions have been shown to be critical for the associated disease pathology in diseases such as Friedreich ataxia (Nakamori and Thornton, 2010; Evans-Galea et al., 2012; Soragni et al., 2014). It has also been suggested that these changes could modulate instability of STRs (Nichol and Pearson, 2002; Gorbunova et al., 2004; Libby et al., 2008; Debacker et al., 2012; Gannon et al., 2012). The Roadmap Epigenomics project (Roadmap Epigenomics Consortium et al., 2015) performed genome-wide epigenetic assays for primary cells and tissue types at unprecedented scale, and offers a timely opportunity to computationally explore the epigenetic context of tri-nucleotide repeats (TNRs) and their association with repeat expansion.

Based on their relative position within a gene, broadly, TNRs can be grouped as “coding” (exonic), and “non-coding” (intronic, intergenic, etc.). The former class includes almost exclusively CAG repeats that lead to polyglutamine stretches in proteins observed in Huntington's disease and most of the spinocerebellar ataxias. In contrast, repeat expansions in introns, 5′ and 3′ UTR regions of genes, are of different types, exemplified by the GAA repeat expansion in Friedreich ataxia, fragile X syndrome and myotonic dystrophy(Lopez-Castel et al., 2010).

A comparison of the triplet repeats (≥6 repeat units) across the human genome revealed that more than 10,000 tandem repeats are present within the genic regions (Willadsen et al., 2013). However, the repeat expansions have been reported in only a handful of cases representing approximately 30 disease conditions, even though in theory all the repeats may have the potential to undergo expansion. This raises the question as to what factors determine the propensity of a given repeat to undergo expansion.

In recent years, experiments on relevant TNR disease model cell types point to an association of heterotransplantation with both coding and non-coding repeat expansions (Dion and Wilson, 2009; Cohen-Carmon and Meshorer, 2012). The formation of heterochromatin appears to be effected via histone modifications as well as DNA methylation at CpG islands at or very near the repeat expansions. The transition into a repressed state is a unifying aspect associated with triplet expansions raising the question, does an epigenetic environment exist that is unique to expanded, disease-associated TNRs? It also raises an interesting possibility of epigenetic features governing the propensity of a given repeat to undergo expansions. We therefore considered whether epigenetic features discerned in normal, healthy cells can help explain and predict if a TNR can, under some circumstance, become unstable and cause disease.

To answer these questions we sought to distinguish between disease-associated TNRs (DA-TNRs), from a set of background TNRs (bg-TNRs; all TNRs, not distinguishing between expanding and those that cause disease) to assess whether we could arrive at common principles. Even though the underlying genes have been cloned for several triplet expansion diseases, a comprehensive dataset with genomic co-ordinates has not been compiled so far, which is essential for the computational exploration of common principles. To achieve this, we first recorded canonical genomic coordinates of DA-TNRs at a base pair level. Having the exact locations to what we refer to as a foreground set, enabled us to precisely link genomic properties to the repeat tract (e.g., histone acetylation and methylation or DNA methylation patterns), and to statistically evaluate their association with repeats *en masse*, i.e., in relation to a background set.

In this paper, we test which epigenetic marks are enriched for (grouped) repeat loci of interest. We develop three complementary statistical tests to analyse the typical epigenetic context of DA-TNRs and compare them with bg-TNRs, stratified by sequence composition and genic location. This stratification allows us to compare the epigenetic patterns statistically, across a spectrum of cell types, that do not harbor expansions (Roadmap Epigenomics Consortium et al., 2015). Specifically, we stratify repeats by their sequence composition (e.g., CAG), their location in the gene (exon, intron, 5′ UTR and 3′ UTR) or a combination of these two features (e.g., exonic CAG). This paper shows that the expansion-prone repeats differ from random set of repeats in their epigenetic profiles, which suggests that certain epigenetic profiles could be associated with an increased propensity for repeats to undergo expansion.

## 2. Materials and methods

### 2.1. Determining the genomic co-ordinates of triplet repeats associated with repeat expansion diseases

We determined the genomic location for 32 known tri-nucleotide repeats associated with disease, relative to human genome build 19 (hg19; UCSC) and the 2009 human reference sequence (GRCh37; NCBI). The position of each disease gene was obtained through the UCSC genome browser and when multiple variants were encountered, the minimum and maximum positions were used. We used RefSeq annotation track on UCSC table browser to generate the gene sequence separated into introns, exons and UTRs. A manual search was carried out to locate each repeat and confirm its position in an exon, intron or UTR region (genic annotation). Where possible, repeat locations were confirmed using the ZIP-seq database of CAG repeats (Xu et al., 2014). ZIP-seq recovered locations for eleven of seventeen CAG DA-TNRs.

The location data for all DA-TNRs are shown in Table 1 but are also made available in the Supplementary Material both as a spreadsheet, which contains references to primary publications, and as a standard BED file, which will allow users to view locations in the UCSC Genome Browser (http://genome.ucsc.edu) and process its content using most genome sequence tools (e.g., BEDTOOLS).

**Table 1:** **Disease-associated tri-nucleotide repeats examined in this study**.

**Disease**	**Disease ID**	**Gene**	**Locus (hg19)**	**Repeat**
**CODING, POLY-GLU REPEATS**				
Spinobulbar muscular atrophy	SBMA	AR	X:66765160	CAG
Huntington's disease	HD	HTT	4:3076604	CAG
Dentatorubral-pallidoluysian atrophy	DRPLA	ATN1	12:7045880	CAG
Spinocerebellar ataxia type 1	SCA1	ATXN1	6:16327213	CAG
Spinocerebellar ataxia type 2	SCA2	ATXN2	12:112037083	CAG
Spinocerebellar ataxia type 3	SCA3	ATXN3	14:92537280	CAG
Spinocerebellar ataxia type 6	SCA6	CACNA1A	19:13318283	CAG
Spinocerebellar ataxia type 7	SCA7	ATXN7	3:63898362	CAG
Spinocerebellar ataxia type 17	SCA17	TBP	6:170870996	CAG
Potassium channel gene	KCNN3	KCNN3	1:154841700	CAG
Amplified in breast cancer 1	AIB1	NCOA3	20:46279816	CAG
**CODING, POLY-ALA REPEATS**				
Soluble programmed death-1	SPD1	HOXD13	2:176957782	GCG
Oculopharyngeal muscular dystrophy	OPMD	PABPN1	14:23790681	GCG
Associated with cleidocranial dysplasia, reduced bone mineral density and bone fracture	CBFA1	CBFA1/ RUNX2	6:45390487	GCG
Holoprosencephaly	ZIC2	ZIC2	13:100637703	GCG
Associated with hand-foot-genital syndrome	HOXA13	HOXA13	7:27238886	GCG
Associated with blepharophimosis syndrome (BPES)	FOXL2	FOXL2	3:138665094	GCG
Early infantile epileptic encephalopathy type 1	EIEE1	ARX	X:25031140	GCG
**CODING, POLY-ASP REPEATS**				
Associated with pseudochondroplasia (PSACH) & multiple epiphyseal dysplasia (MED)	COMP	COMP	19:18896872	GAC
**NON-CODING REPEATS**				
Associated with Fuchs' endothelial corneal dystrophy (FECD)	CTG18.1	TCF4	18:53253401	CAG
Myotonic dystrophy type 1	DM1	DMPK	19:46273199	CAG
Friedreich ataxia	FRDA	FXN	9:71652203	GAA
Spinocerebellar ataxia type 8	SCA8	ATXN8OS	13:70681356	CAG
Spinocerebellar ataxia type 12	SCA12	PPP2R2B	5:146258400	CAG
Huntington's disease-like 2	HDL2	JPH3	16:87637894	CAG
Not currently Associated with a phenotypic abnormality	MAB21L1	MAB21L1	13:36050618	CAG
Candidate gene for autism spectrum disorders (ASDs)	RELN	RELN	7:103629939	GCG
Fragile X syndrome/Fragile X tremor ataxia syndrome	FRAXA/ FXTAS	FMR1	X:146993569	GCG
Folate-sensitive fragile site FRA10A	FRA10A	FRA10AC1	10:95462158	GCG
Fragile X syndrome	FRAXE	FMR2	X:147582153	GCG
Fragile X syndrome	FRAXF	FAM11A	X:148713314	GCG
Folate-sensitive fragile site FRA11B	FRA11B	CBL2	11:119077000	GCG

Columns include disease, disease ID/abbreviation, causal or associated gene for the disease, the genomic location of the repeat, and the repeat sequence. The genomic location is based on the reference hg19 and includes chromosome and starting position. See Supplementary Table 1 for references to supporting literature.

We made use of both computationally deciphered TNRs from genome sequences as well as experimentally confirmed TNRs as a statistical background (bg-TNRs) to DA-TNRs. The “computationally deciphered” background was constructed by scanning the whole human genome using Tandem Repeats Finder (Benson, 1999) to identify *all* repeats which share either genic annotation or sequence repeat unit with one or more DA-TNRs. Zinc finger protein-based immunoprecipitation and sequencing (ZIP-seq) has allowed for genome-wide analysis of TNRs leading to development of a database of CAG repeats located in genes throughout the human genome (Xu et al., 2014). The ZIP-seq database has higher sensitivity than Tandem Repeats Finder and makes up our experimentally determined background of TNRs of length ≥3 repeat units.

### 2.2. Selection of epigenetic marks and data sets

To start with, we selected a subset of genomic signatures based on their presumed or demonstrated roles in chromatin structure, DNA replication or repair. Some of these marks have also been associated with repeat instability (Libby et al., 2008; Dion and Wilson, 2009; Nakamori and Thornton, 2010; Datta et al., 2011; Cohen-Carmon and Meshorer, 2012; Debacker et al., 2012; Volle and Delaney, 2012). The Roadmap Epigenomics Project (Roadmap Epigenomics Consortium et al., 2015) and the ENCODE Project (ENCODE Project Consortium, 2012) encompass genome-wide high-quality data sets, which can be utilized to answer specific questions statistically. We selected nine genome-wide assays from the Roadmap Epigenomics data repository, all of the histone modifications designated as core epigenetic markers (available as ChIP-seq data for 111 tissues; see Table 2) and additional markers directly or indirectly implicated in repeat instability (available in 37 or more tissues; see Table 2), allowing an assessment that was relatively unbiased in regards to tissue. We complemented this dataset with genome-wide assays from the ENCODE data repository, covering signatures from human embryonic stem cells (hESC). For the ENCODE data, we used several markers for euchromatin, heterochromatin and nucleosome occupancy, including DNase I hypersensitivity assays (Crawford et al., 2006) and FAIRE (Giresi et al., 2007). We also used a large number of ChIP-seq data sets (Johnson et al., 2007) to probe binding events on DNA and chromatin for a large number of relevant proteins and their modifiers, including histone deacetylase (HDAC), the chromatin insulator CTCF, and an array of histone tail modifications.

**Table 2:** **Epigenetic marks analyzed in this study**.

**Assay**	**Description**	**Cell type**	**Source**
H3K4me1	A mark of regulatory elements associated with enhancer regions	111	ENCODE Project Consortium, 2012; Roadmap Epigenomics Consortium et al., 2015
H3K4me3	A mark of regulatory elements associated with promoter regions	111	ENCODE Project Consortium, 2012; Roadmap Epigenomics Consortium et al., 2015
H3K9me3	A repressive mark associated with heterochromatin regions	111	ENCODE Project Consortium, 2012; Roadmap Epigenomics Consortium et al., 2015
H3K27me3	A repressive mark associated with Polycomb complex activity	111	ENCODE Project Consortium, 2012; Roadmap Epigenomics Consortium et al., 2015
H3K36me3	An elongation mark associated with transcribed regions	111	ENCODE Project Consortium, 2012; Roadmap Epigenomics Consortium et al., 2015
H3K9ac	A mark of active regulatory elements associated with increased activation of enhancer and promoter regions	44	ENCODE Project Consortium, 2012; Roadmap Epigenomics Consortium et al., 2015
H3K27ac	A mark of active regulatory elements associated with increased activation of enhancer and promoter regions	82	ENCODE Project Consortium, 2012; Roadmap Epigenomics Consortium et al., 2015
DNAm	A mark associated with chromatin structure, silencing gene expression and maintaining stability in repetitive DNA	40	Robertson, 2002; ENCODE Project Consortium, 2012; Roadmap Epigenomics Consortium et al., 2015
DNase	Denoting regions of accessible chromatin commonly associated with regulatory DNA regions	37	ENCODE Project Consortium, 2012; Roadmap Epigenomics Consortium et al., 2015

The columns include the standard abbreviation of the epigenetic mark, its biological description, the number of cell types explored in this study along with the data source.

### 2.3. Statistical enrichment analysis

**Figure 1:**
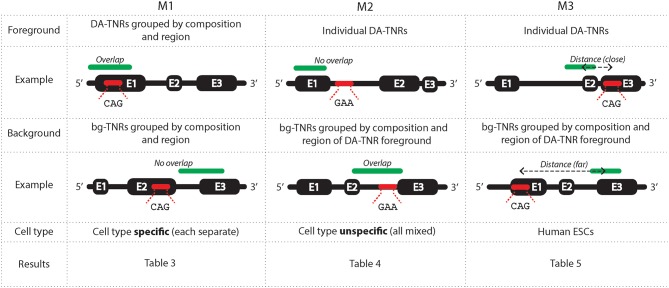
**A graphical representation of the three methods used to measure an enrichment of epigenetic environment around DA-TNRs**. M1 measures an association between a group of DA-TNRs and an epigenetic mark on basis of overlap within each cell type. M2 measures an association between a specific DA-TNR and an epigenetic mark on basis of their overlap when cell type is ignored. M3 uses a distance from the centre of the repeat to the centre of the epigenetic mark to measure enrichment. Each method makes different use of assays from available cell types, foregrounds and statistical tests to measure enrichment.

We developed three methods M1-M3 (summarized in Figure 1) to analyse the epigenetic states around tri-nucleotide repeat regions throughout the genome. We labeled each foreground and background repeat with genomic region (exon, intron, 5′ UTR and 3′ UTR) and canonical tri-nucleotide repeat sequence composition (amongst DA-TNRs there were only four found, of ten possible: GAA.TTC, GAC.GTC, CAG.CTG, and GCG.CGC). Grouped by composition and/or region, DA-TNRs could be compared against similar repeats in the genome. We considered the association of the epigenetic marks and their overlap in individual cell types across repeats grouped by composition and/or region (M1), the overlap of epigenetic marks with specific individual repeats but with no attention to cell type (M2), and the distance of an epigenetic mark from a repeat of interest (M3). These three independent measures provide statistical robustness for assessing the enrichment of the marks around expanded repeats.

M1 determines the enrichment of an epigenetic mark overlapping with a *group* of DA-TNRs. A contingency matrix is created using counts of grouped DA-TNRs versus bg-TNRs which overlap versus do not overlap an epigenetic mark. Both DA- and bg-TNRs are stratified based on applicable combinations of their genic annotation (exon, intron, 5′ and 3′ UTR) and/or repeat unit sequence (CAG, GCG, GAC, and GAA). For a TNR (DA-TNR or bg-TNR) to be in a histone modified state, M1 requires that either the start or the end point is within the called ChIP-seq broad peak. For a TNR to be DNA methylated, M1 requires that its start or end point is within 200 base pairs of a methylated CpG. This more permissive criterion accommodated that DNA methylation sites are shorter than histone broad peaks. We also used DNase-seq narrow peaks, and they were required to overlap with the TNR, to be counted as a match.

M1 uses the Fisher Exact test on the contingency matrices to assign a *p*-value for the resulting hypergeometric distribution of counts, measuring the support for the over- or under-enrichment of the epigenetic mark for the defined group of DA-TNRs. (Over-enrichment means that DA-TNRs overlap with the epigenetic mark to a greater extent than bg-TNRs. Under-enrichment means that fewer DA-TNRs overlap with the mark than expected from the counts of overlaps with bg-TNRs.) This method is repeated independently for all different cell types and epigenetic assays. We note the percent of cell types in which the epigenetic mark is found overlapping with the group DA-TNRs to an extent that cannot be explained by comparing against a stratified but otherwise non-specific set of TNRs. This test lacks statistical power for smaller groups of TNRs, e.g., there is only one GAA DA-TNR, and four CAG DA-TNRs annotated within 5′ UTRs. We left *p*-values uncorrected for multiple tests to discern trends across all epigenetic maps for the same mark, or across sub-sets of maps (say, for specific groups of cell types).

M2 evaluates the enrichment of an epigenetic mark overlapping a specific DA-TNR but across all cell types, relative all bg-TNRs with identical sequence and genic annotation, also sampled indiscriminatively from all cell types. The frequencies accrued from bg-TNRs were used with a binomial test to ascertain the cumulative probability of observing the actual number (or more extreme) of overlaps between the specific DA-TNR and the given epigenetic mark, across the now de-identified cell types. The assumption that the epigenetic status is independent of cell type, implicit in M2, enables us to look at each individual DA-TNR statistically, but this violates the understanding that cell fates are reflected in epigenetic states (Roadmap Epigenomics Consortium et al., 2015). To compensate for multiple tests and this assumption, the *p*-value is corrected conservatively before reported.

M3 evaluates for a single DA-TNR an enrichment based on the distance to a given epigenetic mark or factor. For M3, a distance was measured from the center of the repeat to the center of the closest region labeled with the mark. We did not stratify on length of repeat as this would reduce the size of groups and therefore the statistical power. While longer repeats increase the chance of overlap with epigenetic marks, DA-TNRs did *not* demonstrate this tendency over that observed in bg-TNRs, which are similarly length-distributed. For each mark, we created a (kernel-estimated) density of log-transformed absolute distances between (any) bg-TNRs and the corresponding, closest occurrence of that mark. In this estimate, we included all bg-TNRs that had identical sequence and genic annotation as the DA-TNR of interest. From this density we computed a *p*-value by inspecting the area. We only looked at the left (shorter distance) tail of the density, which means we only assigned a *p*-value to the DA-TNR of interest if *proximity* of mark was enriched.

## 3. Results

### 3.1. Expansion-prone triplet repeats have distinct epigenetic features

To compare the features of the disease-associated repeats, we first compiled their reference genomic positions as described above (see Table 1; Supplementary Table 1) and the positions for a stratifiable background set. To assess the differences between the DA-TNRs and bg-TNRs, we probed the enrichment of histone modifications, DNA methylation and DNase I hypersensitivity using the Roadmap Epigenomics project data set (Roadmap Epigenomics Consortium et al., 2015) available for multiple cell types. We generated epigenetic maps for all cell types. Each map identifies all locations of that epigenetic mark that overlap with a TNR in the cell type. The analysis below thus involves a total of 758 epigenetic maps (see Table 2).

Using the M1 method described earlier, for each epigenetic map, we established the significance of the overlap of the epigenetic mark and each grouped DA-TNR, independently for each cell type. Table 3 shows the percentage of different cell types in which DA-TNRs are either over- or under-enriched at *p* ≤ 0.05 for the tested epigenetic mark. (A small *p* indicates that the overlap established in bg-TNRs, fails to explain the overlap between an epigenetic mark and DA-TNRs for the epigenetic map.) In all our tests, the same trends are seen with either Tandem Repeats Finder or ZIP-seq backgrounds. We provide complete results for both backgrounds in the Supplementary Tables and have focused on results based on Tandem Repeats Finder below.

**Table 3:** **Percent of cell types that display over- or under-enrichment for specific epigenetic marks around DA-TNRs per M1 (see Figure 1) where TNRs are grouped by either sequence and/or genic region**.

**Assay**	**Sequence/Region**											
	**Any/Exon (21)**	**Any/Intron (9)**	**Any/5′UTR (11)**	**Any/3′UTR (7)**	**CAG/Any (14)**	**GCG/Any (7)**	**CAG/Exon (12)**	**CAG/Intron (6)**	**CAG/5′UTR (4)**	**CAG/3′UTR (6)**	**GCG/5′UTR (6)**	**GAA/Intron (1)**
**ACTIVE MARKS**												
H3K4me1	2	62	7	1	26	10	5	16	7	1	13	0
H3K4me3	65	100	46	0	95	0	53	82	77	52	0	7
H3K9ac	36	97	3	0	75	0	28	53	53	28	^*^6	0
H3K27ac	17	89	4	0	70	^*^6	18	32	48	12	^*^9	0
*Any*	32	95	32	1	69	1	17	33	40	6	1	0
**REPRESSIVE MARKS**												
H3K9me3	4	10	7	6	^*^8	17	1	5	^*^3	1	20	0
H3K27me3	2	23	20	2	1	32	1	7	^*^1	0	51	0
H3K36me3	0	4	^*^11	0	12	2	0	1	14	1	2	0
*Any*	0	7	3	3	5	7	1	3	5	1	8	0
**DNA METHYLATION**												
DNAm	68	100	50	3	90	5	65	85	85	33	3	98
**DNA ACCESSIBILITY**												
DNase	46	100	41	0	92	0	41	57	65	38	^*^3	5

A row represents a specific epigenetic mark, or when marked “Any,” the union of all marks listed immediately above. Each column represents DA-TNRs grouped by sequence and/or genic region (as indicated) with the absolute number of DA-TNRs in brackets. For each epigenetic mark and DA-TNR group, the percentage of cell types containing a statistically enriched epigenetic mark at p ≤ 0.05 is shown (over-enrichment by default; under-enrichment is marked with asterisk; if both over- and under-enrichment were found for the same epigenetic mark, only the dominant type is shown). bg-TNRs were predicted by Tandem Repeats Finder and stratified by sequence and/or genic region as per DA-TNRs.

Our analyses revealed that DA-TNRs are more DNA methylated than bg-TNRs are, in a majority of cell types (see Table 3). This trend could be observed in both intronic and exonic tandem repeats, in general for CAG repeats, and even for the sole intronic GAA repeat for Friedreich ataxia. (For FRDA, only one cell type “Brain angular gyrus” is not enriched for DNA methylation.) Hence, it appears that positive methylation status can be attributed to many DA-TNR loci (see Table 2). However, repeats of either GCG or GAC, or those that fall in 3′ UTRs (see also Supplementary Table 1 “M1 DNAm”) form a group of exceptions to this trend, amongst known repeat expansion diseases. For all other tests, the average number of cell types which are enriched for DNA methylation at DA-TNRs exceeds 80%. This is interesting because DNA methylation is also observed in many repeat disease models, in particular upstream of the repeat locus (Dion and Wilson, 2009; Kumari and Usdin, 2009).

Analysis of the histone markers revealed some surprising aspects. We found active histone marks such as H3K4me3, H3K4me1, H3K27ac, and H3K9ac are routinely enriched around locations of many groups of DA-TNRs, in particular those involving the repeat sequence CAG. To quantify their collective enrichment, we refer to the union of all sites for each active mark. Here, on average 27% of all cell types, across all sequence/region permutations, are enriched for active marks (see Table 3, row marked “Any” under section “Active marks”). This was strongest for CAG and intronic repeats, and weakest for 3′ UTR and GCG repeats. This is in contrast to disease models, where expanded TNR loci are associated with silenced chromatin (Dion and Wilson, 2009; Cohen-Carmon and Meshorer, 2012). Indeed, in our analysis, the union of repressive marks displayed almost complete lack of enrichment (see Table 3, row marked “Any” under section “Repressive marks”). That DA-TNRs are enriched for active marks prior to expansion suggest that they are lost in that process. We also note that DNase I is statistically associated with DA-TNRs, with an average of near 60% of cell types having CAG DA-TNRs marked by DNase I at *p* < 0.05, across all genic regions (Table 3).

### 3.2. Few epigenetic markers display a significant enrichment for positional overlap with disease associated triplets

To assess the epigenetic association of each individual DA-TNR, we developed M2 (see Section 2) that compares the frequency of overlap of the DA-TNR and the set of all bg-TNRs with the same genic regions and sequence composition for a given epigenetic mark. For example, the DA-TNR on chromosome X, at locus 66765160 (hg19) is a CAG repeat that is annotated as both exonic and 3′ UTR (see Table 4 - AR). So, we define as background the set of 36 CAG bg-TNRs that are both exonic and 3′ UTR (according to NCBI-RefSeq). The bg-TNRs were then used to determine the frequency of overlap with the epigenetic mark (within 200 base pairs) across *all* cell types available for it.

**Table 4:** **Over- and under-enriched epigenetic marks identified by M2 for individual DA-TNRs (see Figure 1)**.

**Gene Name**	**Locus**	**R#**	**Seq**	**E**	**I**	**5**	**3**	**H3K4me1**	**H3K4me3**	**H3K9me3**	**H3K36me3**	**H3K27me3**	**H3K9ac**	**H3K27ac**	**DNAm**	**DNase**	**Sample**
**CODING, POLY-GLU REPEATS**																	
AR	X:66765160	34	CAG	•			◦	⇑	⇑		⇓	⇑			↑		36
HTT	4:3076604	21	CAG	•					⇑		↓	↓	↑	⇑		⇑	427
ATN1	12:7045880	20	CAG	•				⇑	⇑		⇑	↓		⇑	↓	↑	427
ATXN1	6:16327213	29	CAG	•	◦			↓	⇓		⇑				↑	↓	45
ATXN2	12:112037083	23	CAG	•	◦	◦		⇓	↑					↑		↑	7
ATXN3	14:92537280	27	CAG	•		◦	◦	↓									3
CACNA1A	19:13318283	13	CAG	•			◦				⇓				⇑		36
ATXN7	3:63898362	10	CAG	•			◦		⇑		⇓		↑	⇑	⇑	⇑	36
TBP	6:170870996	47	CAG	•							⇑	⇓			↓		427
KCNN3	1:154841700	17	CAG	•				⇑	⇑		⇓	⇑			↑		427
NCOA3	20:46279816	29	CAG	•				↓	⇓		⇑	↓			↓		427
**CODING, POLY-ALA REPEATS**																	
HOXD13	2:176957782	15	GCG	•				⇑				⇑	↓	⇓			994
PABPN1	14:23790681	7	GCG	•	◦			⇓				⇓		⇑			124
RUNX2	6:45390487	15	GCG	•													994
ZIC2	13:100637703	18	GCG	•					⇓			⇑		↓			994
HOXA13	7:27238886	14	GCG	•				⇑		↑		⇑		↓			994
FOXL2	3:138665094	14	GCG	•				⇑				⇑					994
ARX	X:25031140	15	GCG	•				↑				⇑		↓			994
**CODING, POLY-ASP REPEATS**																	
COMP	19:18896872	7	GAC	•				↑			↓	⇑					478
**NON-CODING REPEATS**																	
TCF4	18:53253401	24	CAG		•			⇑	⇑				⇑	⇑		⇑	760
DMPK	19:46273199	20	CAG				•	↑	⇑		↓		↑	⇑		⇑	89
FXN	9:71652203	6	GAA		•			⇑	⇑	↓			⇑	↑	⇑		3664
ATXN8OS	13:70681356	15	CAG	◦			•			⇑	↓	⇑		⇓	⇓		48
PPP2R2B	5:146258400	11	CAG		◦	•			⇑			⇑					30
JPH3	16:87637894	14	CAG		◦			⇑	⇑			⇑			⇑		785
MAB21L1	13:36050618	19	CAG		◦	•		↑	⇑	↑		⇑					30
RELN	7:103629939	8	GCG			•						⇑		⇓			1354
FMR1	X:146993569	20	GCG	◦		•	◦	↓									13
FRA10AC1	10:95462158	8	GCG		◦	•											237
FMR2	X:147582153	19	GCG			•								⇓			1354
FAM11A	X:148713314	12	GCG			•											1354
CBL2	11:119077000	11	GCG			•											1354

Columns include position in the reference genome (hg19; chromosome and locus), repeat tract length [R#] and [Seq]uence unit, if it occurs in an [E]xon (coding), [I]ntron, [5]′ or [3]′ UTR regions (as annotated by RefSeq denoted by ◦; as reported in literature denoted by •). Enrichment of epigenetic marks is based on multiple cell types from the Roadmap Epigenomics project and are assumed to be independent of cell type, relative bg-TNRs grouped by identical genic region and sequence composition. Arrows indicate over- (blue arrow) or under- (red arrow) enrichment, at a corrected p ≤ 10^–5^ (single arrow) or p ≤ 10^–10^ (double arrow). The sample size of the background is shown in the column “Sample.” The lines separate poly-Gln, poly-Ala, poly-Asp and non-coding repeats. See Supplementary Table 1 for disease identifiers. DNAm is DNA methylation.

For a specific epigenetic mark, we then asked the question: can we explain the frequency of overlap between its occurrence and that single DA-TNR locus across the cell types given the distribution of observations in the corresponding set of bg-TNRs? M2 uses the binomial test to evaluate if the DA-TNR has a significantly different signature–by counting the number of cell types in which it overlaps with the epigenetic mark. The test makes two simplifying assumptions: that observations in different cell types are independent, and that relationships between an epigenetic mark and DA-TNRs can be found even when the type of cell is disregarded. The latter violates our biological understanding but allows individual DA-TNRs to be profiled. With all cell types included in the test, the *p*-values were corrected for multiple tests; the marks that were either over- or under-enriched for the DA-TNR are shown in Table 4. (In Table 4 arrows point up for *more*, arrows point down for *less* cell types than expected.)

Our analyses revealed variation in the epigenetic state for individual DA-TNRs across cell types does vary. Many, but not all CAG DA-TNRs are enriched with H3K4me3, and less consistently with H3K9ac and H3K27ac. DNA methylation status is not as clear for individual repeats, as it was when DA-TNRs were viewed in groups. Four (of 32) individual DA-TNRs (all exonic CAG) are under-enriched for methylation. The FXN TNR is flagged as significantly DNA methylated, together with H3K4me1, H3K4me3, H3K9ac, and H3K27ac. In general, this still suggests that the epigenetic state in disease models (Al-Mahdawi et al., 2008; Dion and Wilson, 2009; Kumari and Usdin, 2009) are distinguishable from healthy cell types not in terms of DNA methylation but in terms of the absence of histone marks related to active and open chromatin (see Table 4).

### 3.3. Enrichment of other epigenetic marks around TNR loci

In addition to markers surveyed by the Roadmap Epigenomics project, we also considered FAIRE (Giresi et al., 2007), which measures DNA accessibility akin to DNase I hypersensitivity, and the binding of factors such as histone deacetylases (HDAC). This data are available from ENCODE (ENCODE Project Consortium, 2012).

We established what factors and markers that are *close* to disease linked TNR loci but not to other, comparable repeat loci. Specifically, we developed M3 (see Section 2) to score co-occurrence in terms of absolute genomic distance, designating a mark as more strongly associated with a locus at an absolute distance *d*, than a locus at a distance exceeding *d* (where “absolute” means that we disregard if the mark is upstream or downstream of the locus). By evaluating enrichment in terms of distances we are able to apply a more sensitive test compensating for the lack of cell types.

To ascertain the significance of the enrichment of each epigenetic mark or factor at loci of DA-TNR, as before, we relied on stratified bg-TNRs. We thus know, for each mark, how close it is in general to a repeat, and could assign a *p*-value to indicate the probability that the distance to the DA-TNR can be explained by chance. Taken together, the distances and *p*-values for the 20 different epigenetic marks and factors, for each DA-TNR locus are provided in the Supplementary Tables.

Table 5 again lists all known DA-TNRs, together with the genomic regions they overlap with, and this time the marks and factors we found to be significantly near to the locus of the DA-TNR. We noticed that many DA-TNRs are enriched for one or more marks, in general those indicating open chromatin, DNA methylation and specific subsets of histone-3 methylation–in agreement with the results rendered by the overlap enrichment.

**Table 5:** **Proximity-enriched epigenetic marks in Human ESCs identified by M3 for individual DA-TNRs (see Figure 1)**.

**Gene name**	**Sequence**	**Region**				**Linked marker(s)**
		**E**	**I**	**5**	**3**	**Closer**
**CODING, POLY-GLU REPEATS**						
AR	CAG	•			◦	H4K20me1
HTT	CAG	•				H3K4me3, H3K9ac
ATN1	CAG	•				H4K20me1
ATXN1	CAG	•	◦			DNAm
ATXN2	CAG	•	◦	◦		DNAm
ATXN3	CAG	•		◦	◦	
CACNA1A	CAG	•			◦	DNAm
ATXN7	CAG	•			◦	CTCF
TBP	CAG	•				
KCNN3	CAG	•				H3K9ac, HDAC2
NCOA3	CAG	•				H2az
**CODING, POLY-ALA REPEATS**						
HOXD13	GCG	•				
PABPN1	GCG	•	◦			H3K36me3, FAIRE
RUNX2	GCG	•				H2az
ZIC2	GCG	•				H3K36me3
HOXA13	GCG	•				DNase
FOXL2	GCG	•				CTCF
ARX	GCG	•				DNase, FAIRE
**CODING, POLY-ASP REPEATS**						
COMP	GAC	•				H4K20me1
**NON-CODING REPEATS**						
TCF4	CAG		•			
DMPK	CAG				•	H4K20me1, HDAC2
FXN	GAA		•			H2az, H3K27ac, H3K4me1, H3K4me2, H3K4me3, H3K9ac, CTCF, DNase
ATXN8OS	CAG	◦			•	H3K4me1
PPP2R2B	CAG		◦	•		H3K27ac, DNase
JPH3	CAG		◦			H3K27me3, H3K79me2, HDAC2
MAB21L1	CAG		◦	•		H4K20me1
RELN	GCG			•		H3K20me1
FMR1	GCG	◦		•	◦	
FRA10AC1	GCG		◦	•		CTCF (x2), H3K4me3, H3K79me2, H3K9ac
FMR2	GCG			•		H3K27me3
FAM11A	GCG			•		
CBL2	GCG			•		

Columns include gene name and sequence unit, if it occurs in an [E]xon (coding), [I]ntron, [5]′ or [3]′ UTR regions (as annotated by RefSeq denoted by ◦; as reported in literature denoted by •), and which epigenetic marks are significantly closer to DA-TNRs, relative bg-TNRs (p ≤ 0.05) in human ESCs.

## 4. Discussion

Across the range of normal, healthy cell types, we note that several groups of DA-TNRs, and individual DA-TNRs appear in H3K4 mono- and tri-methylated regions; in H3K9 and H3K27 acetylated regions (in particular CAG-repeats; see Table 3), all of which are active marks and indicators of open chromatin (Ernst et al., 2011). We also note that most DA-TNRs when grouped are DNA methylated or at least close to methylated CpGs (see Table 3). The latter observation is generally in line with what is also observed in disease tissue, but the former is not. We generally saw the same trends when using ZIP-seq data as statistical background (see Supplementary Tables). Below we make explicit reference to results based on bg-TNRs identified using Tandem Repeats Finder, because they also cover non-CAG repeats.

### 4.1. Interpreting the enrichment of epigenetic marks

The enrichment tests above indicate which cell type specific epigenetic marks differ in their distribution around grouped DA-TNRs when compared to a stratified background. This is evidence that DA-TNRs have epigenetic features that are relatively uncommon for stable TNRs. Statistically over- or under-enriched factors in healthy tissues are key targets for future analyses as they may be altered to either *cause* expansion, or as an *effect* of expansion.

#### 4.1.1. Chromatin structure around DA-TNRs

DNase I or FAIRE are effective markers for DNA accessibility, and are often used to probe the chromatin packaging state. In normal, healthy cells DA-TNRs tend to occur at loci overlapping or proximal to DNase I hypersensitive tracts meaning that DNA is accessible, as illustrated by Table 3 for groups of DA-TNRs and Tables 4, 5 for individual DA-TNRs. FAIRE was proximity-enriched at two DA-TNRs in hESC (see Table 5).

CTCF is a zinc-finger DNA binding protein that plays a role in chromatin insulation, transcriptional regulation and genomic imprinting. It affects higher order chromatin structure and could therefore play a role in repeat instability, particularly in the case of a binding site located 3′ to CAG repeats. Libby et al. (2008) demonstrated this influence on stability at the SCA7/ATXN7 locus and four other CAG repeat loci. We observe that SCA7 and three other DA-TNRs have a CTCF binding site significantly closer than expected by chance in hESC (see Table 5 and Supplementary Tables).

#### 4.1.2. Absence of repressive histone marks in DA-TNRs

Three histone modifications (H3K27me3, H3K9me3, and H4K20me1) are associated with a repressive or heterochromatic environment. These marks were not prevalent around DA-TNRs in normal cell types. The lack of repeats containing these marks in the 758 epigenetic maps from the Roadmap Epigenomics project and over 20 maps extracted from ENCODE's hESC (human embryonic stem cell) is in contrast with observations in disease models (Al-Mahdawi et al., 2008; Dion and Wilson, 2009; Kumari and Usdin, 2009; Cohen-Carmon and Meshorer, 2012). H3K27me3 and H3K36me3 are repressive marks associated with disease models, but we failed to demonstrate a general co-occurrence with DA-TNRs. They were over-enriched in a small number of tissues and also significantly close to very few DA-TNRs, but they also appeared to be under-enriched in a few instances (see Table 3, section “Repressive marks”; also see Table 5). H3K9me3 is also a repressive mark associated with repetitive elements and constitutive heterochromatin (Al-Mahdawi et al., 2008), but we failed to find a clear association with DA-TNRs, with the exception of a weak over-enrichment with GCG TNRs at 5′ UTR (see Table 3). H4K20me1 has been linked to repression and transition to heterochromatin and a number of biological processes including DNA damage repair and DNA replication (Jørgensen et al., 2013). It was only available for the ENCODE data set, but our analysis indicated some support for linking it with DA-TNRs. Six individual DA-TNRs showed significantly closer H4K20 mono-methylation than what would be expected from observing bg-TNRs.

#### 4.1.3. DNA methylation

DNA methylation plays a silencing role in gene expression, chromatin structure and maintaining genome stability in repetitive DNA (Robertson, 2002). DNA methylation patterns have been shown to be very specific to cell types (Varley et al., 2013) while histone modifications reflect changes in gene transcription across cell types (Koch et al., 2007). Both M1 and M3 consider patterns of overlap and proximity in a cell type specific manner, and are therefore capable to detect both methylation and histone modification patterns. On the other hand, M2 is only seeing an epigenetic signal for repeat loci if it is independent of cell type. We therefore caution against over-interpreting the enrichment of DNA methylation observed by M2. This problem with M2 affects DNA methylation patterns more than histone modifications, as methylation is more cell type specific than histone modifications.

**Figure 2:**
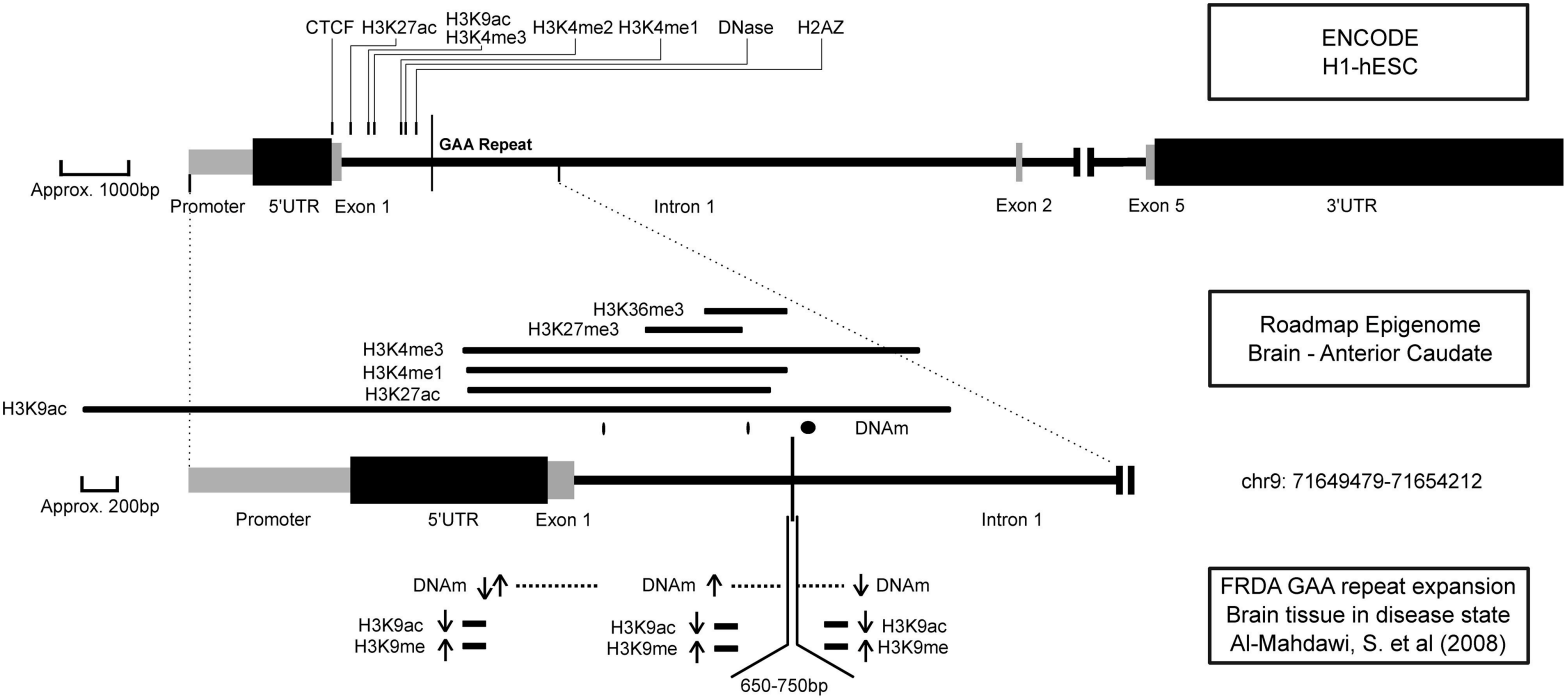
**The epigenetic environment of FRDA**. A representation of epigenetic marks with their location around the Frataxin gene (NM_000144) across three experimental data sets (named in the right margin). Upper panel: Marks are displayed if they are significantly close to the GAA repeat according to M3. Middle panel: The full-width peaks for each mark around the GAA repeat according to the Roadmap Epigenome for healthy brain tissue. Lower panel: Changes in epigenetic marks as observed in brain tissue, based on differences between normal and diseased state (Al-Mahdawi et al., 2008). Locations where DNAm and histone assays were performed are shown. Arrows indicate a significant increase or decrease in methylation and histone binding at these locations. The size of the GAA repeat expansion in the disease state is also indicated.

DNA methylation occurs at higher levels in CpGs upstream of the GAA repeat and in some CpGs in the 5′ UTR in FXN (Evans-Galea et al., 2012), and with multiple other repeat expansion models (Dion and Wilson, 2009). Several other ataxia-related phenotypes (SCA1, SCA2, and SCA6) showed close methylation patterns (see Table 4). It is striking that DNA methylation is statistically enriched at sites of DA-TNRs compared to genomically similar sites in a majority of tissues, clearly suggesting that it is a feature of disease, but one which fails to directly explain the transition of a repeat from a normal to an expanded state. Speculatively, a transient state of de-methylation *could* provide an explanation, as reduced methylation levels are known to promote both contractions and expansions in selected cell lines (Gorbunova et al., 2004). The DNAm states in Figure 2 show that DNA methylation is site specific with different locations around the GAA repeat showing both increases and decreases in DNA methylation of CpGs. This variation seconds the idea of transience in the methylation state around unstable TNRs.

The key hypothesis that can be drawn from the above observations is that DA-TNRs are located in an epigenetic environment enriched for markers of open chromatin and active regulation making them susceptible to histone modifications introducing repressive marks, alterations to the chromatin landscape, transcience in methylation state and, ultimately, repeat expansion mutations.

Overall these findings suggest that transcriptionally active regions that harbor repeats may be more prone to DNA mutations thus increasing the propensity of repeats to undergo expansions. For instance, it has been shown that transcriptionally active chromatin recruits homologous recombination at DNA double strand-breaks (Aymard et al., 2014). Homologous recombination is one such mechanism that has been previously suggested to contribute to the expansion of repeats (Zhou et al., 2001).

### 4.2. Application of results to a disease model

Friedreich ataxia (FRDA) is a loss-of-function disease caused by an expanded intronic GAA repeat in the Frataxin (FXN) gene, and in our analysis it displayed the greatest number of epigenetic marks in its distance profile (see Table 5). Figure 2 indicates the approximate location of these marks as assayed in hESCs and brain tissue, and as observed in disease models, from the data we analyzed and the experimental literature, respectively. The marks from hESCs and brain tissue contribute to an open chromatin environment and indicate a proximal active promoter (Ernst et al., 2011). In combination, they show that epigenetic marks identified as significant in hESCs are also observed near the GAA repeat in a tissue-specific sample including H3K9ac, H3K27ac, H3K4me1, and H3K4me3. An alteration to histone-3, lysine-9, and lysine-27 from acetylation to repressive tri-methylation modifications would cause a transition to a repressive heterochromatic environment (Hahn et al., 2010). In the third set of factors in Figure 2 from diseased brain tissue, the alteration from H3K9ac to H3K9me can be observed. Significant variations in methylation, a loss of H3K9 acetylation and increase in H3K9 methylation around the GAA repeat and 5′ UTR all indicate a repressive heterochromatic environment in the disease state. Based on our statistical analyses of epigenetic marks around the repeat, specific hypotheses can be drawn to address this transition. For example, this is consistent with the findings that suggest that the inhibitors of histone deacetylase increase FXN expression levels (Herman et al., 2006). It would be interesting to assess whether blocking histone methyl transferase to prevent H3K9ac and H3K27ac would promote the stability of the GAA repeat.

## 5. Conclusion

We note that DA-TNRs are placed in regions ranging from 5′ to 3′ UTR, in exons and introns. While epigenetic states tend to vary widely between such regions and tissues, we have observed consistency of epigenetic states around DA-TNRs that are not associated with bg-TNRs. Since our comparisons are stratified, this consistency of epigenetic state can hypothetically be attributed to the shared biological circumstance–that of expanding under some yet unknown condition.

By using the breadth and depth of DNA and chromatin state data we were able to determine statistical enrichment of epigenetic marks around a repeat expansion locus and to provide qualified support for observations relevant to mechanisms behind repeat expansion itself. However, there are several caveats to what can come out of such analyses. While research has been conducted to investigate the changes in the surrounding genomic context of repeats, how the chromatin structure directly relates to repeat, is still unresolved. Cultured cells and transgenic models provide clues to epigenetic states of repeat expansions, but it is well acknowledged these cells can develop non-physiological epigenetic profiles (Al-Mahdawi et al., 2008). This paper develops a complementary resource for understanding epigenetic states under normal conditions, and a group of methods that when disease tissue becomes available enable the analysis of the epigenetic states linked to repeat instability.

Our statistical analyses brings the scale of epigenetic data offered by recent technologies to bear, to identify a handful of what appear significant epigenetic markers that should be assayed with priority in disease tissues, in particular DNA methylation, H3K4me3, H3K9, and H3K27 acetylation, DNase I hypersensitivity and HDAC. We also note that our analyses support the development of signatures that can be used to identify new repeat loci, with potential to expand.

## Author contributions

AE, PV, and MB collated and evaluated literature data and evidence for DA-TNRs, performed and interpreted analyses. MC, BC, and SB contributed to analyses. AE and MB drafted the manuscript; all authors wrote and approved of the manuscript. SB and MB designed and supervised the study.

### Conflict of interest statement

The authors declare that the research was conducted in the absence of any commercial or financial relationships that could be construed as a potential conflict of interest.
